# Identification and characterization of high‐yielding, short‐duration rice genotypes for tropical Asia

**DOI:** 10.1002/csc2.20183

**Published:** 2020-08-03

**Authors:** Phyo L. P. Won, Hongyan Liu, Niño P. M. Banayo, Lixiao Nie, Shaobing Peng, Mohammad R. Islam, Pompe Sta. Cruz, Bertrand C. Y. Collard, Yoichiro Kato

**Affiliations:** ^1^ Department of Agronomy Yezin Agricultural Univ. Nay Pyi Taw 15013 Myanmar; ^2^ College of Tropical Crops Hainan Univ. Haikou Hainan 570228 China; ^3^ International Rice Research Institute DAPO Box 7777 Metro Manila 1301 the Philippines; ^4^ College of Agriculture and Food Science Univ. of the Philippines Los Baños Laguna 4301 Philippines; ^5^ College of Plant Science and Technology Huazhong Agricultural Univ. Hubei 430070 China; ^6^ Department of Primary Industries Yanco Agricultural Institute Yanco Yanco NSW 2703 Australia; ^7^ Graduate School of Agricultural and Life Sciences Univ. of Tokyo Tokyo 1138657 Japan

## Abstract

Previous efforts to increase the yield of tropical rice (*Oryza sativa* L.) have focused on medium‐duration varieties. However, there is increasing demand for high‐yielding short‐duration varieties that can adapt to intensified cropping systems and climate change. Our goal was to identify physiological traits associated with high yield in elite short‐duration genotypes suitable for tropical Asia. We conducted field experiments in five consecutive growing seasons at the International Rice Research Institute, the Philippines. We selected genotypes in the first two seasons, then performed a detailed characterization of the most promising genotypes in the following three seasons. Of the 50 advanced‐generation genotypes, three had consistently high yield and early maturity, with yields 11 to 38% higher than that of ‘IRRI104’ (‘IR50404‐57‐2‐2‐3’), a short‐duration variety that is widely grown in Southeast Asia. These genotypes were 20 to 32 cm taller than IRRI104. We found that for grain growth, low source capacity, defined as stem nonstructural carbohydrates at heading plus biomass accumulation after heading, was the major factor for the low yield of IRRI104. Although sink capacity (spikelets m^−2^ × grain weight) in the promising genotypes was comparable to that of IRRI104, they had a 25 to 53% higher source–sink ratio (source capacity/sink capacity) than IRRI104, which was attributed to larger leaf area and greater biomass accumulation during the grain‐filling stage. This result suggests that slight changes in plant development to promote height combined with increased leaf area around heading would improve the yield of short‐duration rice varieties in tropical Asia.

**Abbreviations**
CGR: crop growth rate
DAT: days after transplanting
DS: dry season
EWS: early wet season
IRRI: International Rice Research Institute
LAI: leaf area index
LWS: late wet season
NAR: net assimilation rate.

## 1 INTRODUCTION

Rice is a key tropical staple food crop and demand for rice is projected to increase as populations continue to grow in Asia and as rice consumption increases rapidly in Sub‐Saharan Africa (Global Rice Science Partnership, 2013). The impacts of climate change are increasingly evident and extreme weather events around the world increasingly threaten food security. As a result, researchers predict a 12 to 14% decrease in rice production by 2050 compared with production in 2000, with tropical Asia being affected most (Wheeler & von Braun, 2013). To mitigate this problem and protect regional food security, it will be necessary to stabilize or increase rice production (Atlin, Cairns, & Das, 2017).

Yield potential, a genotype‐specific trait, represents a cultivar's maximum yield when it is grown in the absence of biotic and abiotic stresses (Kropff, Cassman, Peng, Matthews, & Setter, 1994). Raising yield potential has therefore been a major goal of breeding efforts in the modern era (Peng & Khush, 2003). Past research has shown that since the 1960s, breeding rice for high yield in Asia frequently targeted semidwarf stature, which increased the harvest index and panicle size, thereby increasing the sink capacity compared with older varieties (Khush, 1995; Peng, Khush, Virk, Tang, & Zou, 2008). The challenge for breeding the semidwarf ideotype since the 1980s at centers such as the International Rice Research Institute (IRRI) was how to find the optimal combination of agronomic traits for medium‐duration varieties with a growth duration of 115 to 120 d (Peng et al., 2008). This approach further increased rice yields by the 2000s (Dingkuhn et al., 2015). As a result of these efforts, most medium‐duration elite genotypes now have a harvest index that approaches the theoretical maximum of 0.55 to 0.60 (Hay, 1995). Another problem is that elite accessions now produce numerous spikelets per unit of area by developing large panicles, with 150 to 200 spikelets per panicle (Peng et al., 2008), in which grain filling is limited by a shortage of nonstructural carbohydrates or concurrent photoassimilation. This has become a bottleneck in recent high‐yielding varieties (Dingkuhn et al., 2015; Yoshinaga, Takai, Arai‐Sanoh, Ishimaru, & Kondo, 2013).

At the same time, climate change has led researchers to pay more attention to whether or not cultivation systems can be adapted to reduce the risk of damage caused by drought, flooding, and saltwater intrusion. One approach is to use short‐duration varieties (Campbell et al., 2016). In rainfed rice ecosystems, varieties with a growth duration of 95 to 105 d can both escape drought at the end of the wet season (Ohno et al., 2018) and permit more intense cultivation, with dryland crops being established in the wet season immediately after the rice harvest to take advantage of the residual soil moisture (Haefele, Kato, & Singh, 2016). Short‐duration varieties also have advantages over longer growth duration varieties. These include less risk of typhoon‐driven lodging and of pest damage (rodents, birds, and insects), combined with the higher sale price for providing the first harvests during a given cultivation season (Xu et al., 2018). In irrigated rice ecosystems, many farmers prefer short‐duration varieties, since they often face serious water shortages late in the dry season. In Cambodia's Mekong Delta and Myanmar's Ayeyarwady (Irrawaddy) Delta, some farmers now produce two rice crops by growing short‐duration varieties during the dry season but leave their fields fallow during the wet season because of the risk of flooding (Fukai & Ouk, 2012).

**Core Ideas**

- Key traits for high yield in short‐duration rice (SDR) were examined.
- New SDR lines yielded 11 to 38% more than a reference variety.
- Breeding SDR should aim to enhance source capacity during grain filling.

Despite increasing demand for short‐duration rice varieties, genetic improvements to achieve high yields have been slow. Previous breeding programs have focused on medium‐duration varieties (Peng & Khush, 2003), as their potential is generally higher than that of short‐duration varieties under optimal conditions (Tirol‐Padre et al., 1996). This is because crop biomass and the associated ability to capture resources (e.g., radiation, nutrients, water) increase with increasing growth duration (Kropff et al., 1994). Currently, IRRI104 is highly popular and is grown on more than 1 million ha in the Mekong Delta and the Philippines (Khush et al., 1995; Ohno et al., 2018; Wang, Velarde, Bona, & Meas, 2012). This IRRI variety, which was released as MTL87 in Vietnam in 1988 and as PSB Rc10 in the Philippines in 1992, is still a favorite choice despite its low yield because of its short growth duration coupled with adaptation to a broad range of growing conditions.

Researchers do not yet know whether or not the abovementioned knowledge related to the semidwarf ideotype and target traits that have been used to approach the theoretical maximum yield in medium‐duration varieties can also be applied to improve short‐duration varieties such as IRRI104. The semidwarf ideotype can increase the harvest index; however, the reduced plant size limits leaf area expansion and biomass accumulation during vegetative growth (Kato & Katsura, 2014). Although short‐duration varieties have a short period of vegetative growth, the reproductive period is similar for all rice genotypes (Kropff et al., 1994). Thus reducing the growth duration of rice genotypes is likely to change the relative importance of (and the balance between) panicle number and size during sink formation. Furthermore, we do not yet know whether the primary limitation on yield arises from sink capacity, source capacity, or a combination of both for short‐duration varieties.

Developing high‐yielding short‐duration varieties will improve our options for responding to climate change and the need for intensified cultivation. To develop such varieties, we must first understand how rice yield correlates with physiological characteristics in elite short‐duration genotypes. Our objective in the present study was to identify the physiological characteristics associated with high yield in short‐duration rice accessions suitable for use in tropical Asia.

## 2 MATERIALS AND METHODS

### 2.1 Experiment 1: Yield of advanced‐generation short‐duration genotypes

We analyzed 50 elite genotypes that were fixed breeding lines from F_8_ or later generations (Supplemental Table S1 and Supplemental Table S2), with 22 genotypes evaluated in all seasons. These genotypes were selected based on superior phenotypic performance and growth duration under nonstressed conditions in the F_5_ and F_6_ generations; breeding was then continued to at least the F_8_ generation before the present study. We included two popular short‐duration varieties: IRRI104 (IR50404‐57‐2‐2‐3) and ‘IRRI123’ (‘IR64683‐87‐2‐2‐3‐3’). We quantified the yield of these accessions at the IRRI farm in Los Baños, the Philippines (14°11′N, 121°15′E, 21 m asl) during the dry season (DS; January–April) and the early wet season (EWS; June–September) of 2015. The soil was an Aquandic Epiaquoll (6% sand, 33% silt, 61% clay) with a pH (H_2_O) of 6.9, 23.7 g total C kg^−1^, 2.0 g total N kg^−1^, 30.0 mg Bray‐II P kg^−1^, 0.87 cmol exchangeable K kg^−1^, and a cation exchange capacity of 39.4 cmol kg^−1^. The air temperature averaged 26.2 °C in DS and 29.0 °C in EWS, solar radiation averaged 16.2 MJ m^−2^ d^−1^ in DS and 16.7 MJ m^−2^ d^−1^ in EWS, and rainfall totaled 150 mm in DS and 737 mm in EWS, according to data from the meteorological station at the IRRI farm.

Genotypes were arranged in 5.6‐ by 1.2‐m plots in a randomized complete block design with two replicates. Transplanting was done on 6 Jan. 2015 in DS and 2 July 2015 in EWS. Two or three 21‐d‐old seedlings were transplanted per hill at a hill spacing of 20 by 20 cm. Plots were fertilized 10 d after transplanting (DAT) at rates of 38 kg N ha^−1^, 22 kg P ha^−1^, and 25 kg K ha^−1^ in DS and 30 kg N ha^−1^, 13 kg P ha^−1^, and 25 kg K ha^−1^ in EWS. In DS, N was also split‐applied at rates of 38 kg ha^−1^ at 24 DAT and 42 DAT and 16 kg ha^−1^ at 62 DAT (130 kg N ha^−1^ in total). In EWS, it was also split‐applied at 30 kg ha^−1^ at 25 DAT and 42 DAT (90 kg N ha^−1^ in total). Paddy water was maintained at a depth of 2 to 3 cm from transplanting to a few days before harvest.

We recorded the number of days to heading from the sowing date until 50% of the panicles had emerged. At maturity (ca. ∼30 d after heading), we measured plant height and panicle number for 10 plants in each plot. All plants in each plot were harvested to determine grain yield, which was adjusted to 14% g H_2_O g^−1^ moisture content.

### 2.2 Experiment 2: Characterization of promising high‐yielding genotypes

Three rice high‐yielding genotypes among the short‐duration genotypes were identified in Experiment 1: ‘IR12A165’ (‘IR91028‐100‐3‐2‐1’), ‘IR13A438’ (‘IR92277‐RIL15‐1‐1‐1‐1’), and ‘IR13A378’ (‘IR92274‐RIL76‐1‐1‐1‐1’). We conducted experiments in irrigated lowlands at the IRRI farm during three periods: DS, EWS, and the late wet season (LWS; September–December) of 2016. The air temperature averaged 27.4 °C in DS, 29.3 °C in EWS, and 27.9 °C in LWS. The solar radiation averaged 15.8 MJ m^−2^ d^−1^ in DS, 15.5 MJ m^−2^ d^−1^ in EWS, and 12.0 MJ m^−2^ d^−1^ in LWS. The rainfall totaled 99 mm in DS, 537 mm in EWS, and 1,151 mm in LWS. The four genotypes were arranged in 5.6‐ by 2.0‐m plots in a randomized complete block design with three replicates in DS and EWS and four replicates in LWS. Fertilization followed the protocols for the dry and wet seasons in Experiment 1. Transplanting dates were 6 Jan. 2016 in DS, 11 May 2016 in EWS, and 2 Sept. 2016 in LWS.

We measured the aboveground biomass, leaf area index (LAI), and plant height at panicle initiation (35 DAT), heading, and physiological maturity by harvesting 10 hills in each plot. The harvested plants were separated into green leaves, stems, and panicles (after heading) and the green leaf area was measured with an LI‐3000 leaf area meter (LI‐COR, Lincoln, NE). The dry weight of each component was determined after oven‐drying for 72 h at 80 °C. The aboveground biomass, as the sum of the dry weight of each component, and the LAI (total leaf area divided by ground area) were calculated. We also calculated the crop growth rate (CGR), net assimilation rate (NAR), and mean LAI for two growth periods: (a) the reproductive growth period from panicle initiation (*t*_1_) to heading (*t*_2_) and (b) the grain‐filling period from heading (*t*_1_) to maturity (*t*_2_). These were calculated as follows, where *t*_1_ and *t*_2_ are dates:

(1) $$\mathrm{CGR}\;=\frac{\mathrm{biomas}s_{t_{2}}-\mathrm{biomas}s_{t_{1}}}{t_{2}-t_{1}};$$

(2) $$\mathrm{NAR}\;=\frac{\mathrm{CGR}}{\mathrm{LA}I_{\mathrm{mean}}};$$

(3) $$\mathrm{LA}I_{\mathrm{mean}}\;=\mathrm{LA}I_{2}-\mathrm{LA}I_{1}/\ln\left(\mathrm{LA}I_{2}\right)-\ln\left(\mathrm{LA}I_{1}\right),$$

where $\mathrm{biomas}s_{t_{1}}$ is biomass at *t*_1_, $\mathrm{biomas}s_{t_{2}}$ is biomass at *t*_2_, CGR is the CGR between *t*_1_ and *t*_2_, LAI_mean_ is the mean LAI between *t*_1_ and *t*_2_, LAI_1_ is LAI at *t*_1_, and LAI_2_ is LAI at *t*_2_.

At heading, we measured the nonstructural carbohydrate concentration in the stem as the sum of the soluble sugar and starch concentrations via the methods of Kato, Collard, Septiningsih, and Ismail (2014). We assayed the soluble sugars with the anthrone reagent (Sigma‐Aldrich Co., MO) and assayed the starch after hydrolysis with amyloglucosidase (Sigma‐Aldrich Co.), followed by a glucose assay with glucose oxidase (Sigma‐Aldrich Co.).

At maturity, we counted the number of panicles in a 4.2‐m^2^ area that we subsequently used to determine the grain yield. We randomly harvested 10 hills to measure the yield components and harvest index (weight of filled grains divided by aboveground biomass). We separated the panicles from the straw by hand‐threshing and separated filled and unfilled spikelets by flotation in tap water. We counted the number of spikelets per panicle, the filled grain percentage, and the 1,000‐grain weight. Next, we manually separated the unfilled spikelets into empty and partially filled spikelets to determine the spikelet sterility percentage [(empty spikelets ÷ total spikelets) × 100]. We quantified the sink–source relationship during grain filling following the method of Morita and Nakano (2011). In summary, we quantified the sink capacity (spikelets m^−2^ × individual grain weight), dry matter accumulation during grain filling, source capacity (stem nonstructural carbohydrates at heading plus dry matter accumulation during grain filling), source/sink ratio (source capacity/sink capacity), and source capacity per spikelet (source capacity ÷ spikelet number).

### 2.3 Statistical analysis

We analyzed the data with STAR version 2.0.1 software and of PBTools version 1.4, which are open‐access programs implemented in the R package (http://bbi.irri.org, accessed 2 June 2020). We conducted ANOVA separately for each season via general linear models to assess the genotypic variation in Experiment 1. We also combined the seasons to assess the effects of genotype, season, and the genotype × season interaction for the 22 genotypes grown in both DS and EWS. When the ANOVA result was significant, differences were compared by Fisher's LSD test, with significance defined as *p* < .05 (^*^ and ^**^ refer to *p* < .05 and *p* < .01 respectively). To assess yield differences among genotypes after eliminating phenological effects, we used analysis of covariance, with days to heading as the covariate. Since the number of replicates differed among the seasons, we assessed the genotype, season, and genotype × season effects via a mixed model and the restricted maximum likelihood estimation approach.

## 3 RESULTS

### 3.1 Experiment 1: Phenotypic evaluation of elite short‐duration rice genotypes

We observed wide variation in days to heading, grain yield, plant height, and panicle number among the advanced‐generation genotypes in both DS and EWS (Table 1). In DS, grain yield was positively correlated with plant height (*r* = 0.38^*^) and days to heading (*r* = 0.40^*^) but not with panicle number. In EWS, when the plant height and panicle number were both higher than in DS, yield was only significantly correlated with days to heading (*r* = 0.56^**^). However, the analysis of covariance detected significant genotypic variation in yield in both seasons after removing the effect of the covariate (i.e., days to heading).

**TABLE 1:** Agronomic performances of advanced short‐duration rice genotypes at the International Rice Research Institute during the dry and early wet seasons of 2015

	Days to heading	Grain yield	Plant height	Panicles
	d	Mg ha^–1^	cm	m^–2^
Dry season (*n* = 36)				
Mean	83^**^	6.33^*^	95^**^	329^**^
Range	78–96	5.23–7.17	84–114	237–408
CV (%)	2.13	7.38	5.83	9.53
Early wet season (*n* = 36)				
Mean	83^**^	7.26^**^	116^**^	476^**^
Range	75–90	5.96–8.54	95–137	381–600
CV (%)	1.80	8.68	5.33	7.70

^*^Significant genotypic variation at the .05 probability level.

^**^Significant genotypic variation at the .01 probability level.

**FIGURE 1:**
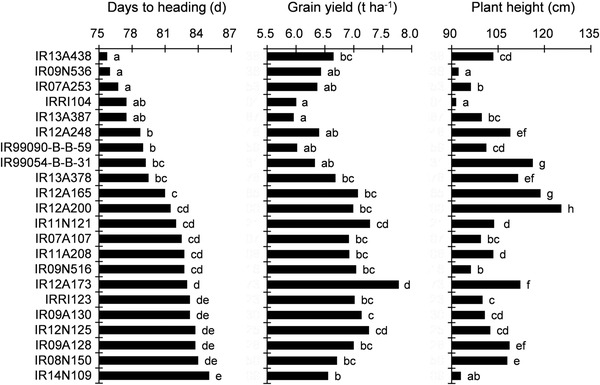
Days to heading, grain yield and plant height for 22 elite genotypes evaluated in both dry and early wet seasons in the short‐duration rice breeding program at the International Rice Research Institute in 2015. Bars labeled with different letters differed significantly at *p* < .05

To identify the short‐duration genotypes with high yield, we compared the 22 genotypes grown in both seasons (Figure 1). Except for IR13A438, yield was higher in genotypes, with 80 to 84 d from sowing to heading, than those with 76 to 79 d to heading, with the peak yield occurring in a genotype with a heading time of 83 d (‘IR12A173’). Nine genotypes had days to heading within 4 d of the value for the reference variety (IRRI104): IR13A438, ‘IR09N536’, ‘IR07A253’, ‘IR13A387’, ‘IR12A248, ‘IR99090‐B‐B‐59’, ‘IR99054‐B‐B‐31’, IR13A378, and IR12A165. Among these genotypes, IR13A438, IR13A378, and IR12A165 had significantly higher yield than IRRI104 (by 11–18%). IRRI104 had the shortest plant height.

### 3.2 Experiment 2: Physiological attributes associated with high yield in the promising genotypes

We chose IR13A438, IR13A378, and IR12A165 as promising accessions because they combined relatively short days to heading with high yield. To identify the physiological characteristics that led to their superior yield performance, we calculated CGR, NAR, and mean LAI (Table 2). During the reproductive stage (panicle initiation to heading), we found no significant genotypic differences in these traits. However, the genotype effects became significant for CGR and mean LAI during the grain‐filling stage; IRRI104 had the lowest CGR. Although genotypic variation was not significant for NAR during either stage, IRRI104 had a much lower NAR after heading, at <77% of the values for the promising accessions. There was no significant genotype × season interaction during either growth stage, suggesting that the promising genotypes showed stable high performance.

**TABLE 2:** Crop growth rate, net assimilation rate, and mean leaf area index (LAI) of elite short‐duration rice genotypes in the early and late wet seasons of 2016

	Crop growth rate		Net assimilation rate		Mean LAI	
	PI^a^ to heading	Heading to maturity	PI to heading	Heading to maturity	PI to heading	Heading to maturity
	g m^−2^ d^−1^				m^2^ m^−2^	
Genotype						
IR12A165	18.2	11.5	6.84	5.05	3.02	2.30
IR13A378	19.5	14.6	8.53	5.97	2.79	2.45
IR13A438	16.8	10.8	6.71	5.45	2.74	2.04
IRRI104	16.1	8.2	7.92	3.87	2.58	2.05
Season						
Early wet season	21.4	13.9	5.32	5.09	4.09	2.65
Late wet season	13.9	8.6	9.68	5.09	1.48	1.77
ANOVA						
Genotype	ns	**	ns	ns	ns	*
Season	*	*	*	ns	**	**
Genotype × season	ns	ns	ns	ns	ns	ns

a PI, panicle initiation; ns, not significant.

^*^Significant at the.05 probability level.

^**^Significant at the.01 probability level.

We observed results similar to those for both DS and EWS in 2015 for days to heading, plant height, and grain yield for the three seasons in 2016 (Table 3). The yields of the three promising genotypes were 29 to 38% greater than that of IRRI104. The difference in days to heading was less than 4 d, except for IR13A378 in EWS, which had 8 d longer than IRRI104. IRRI104 plants were 20 to 32 cm shorter than plants in the other accessions. The aboveground biomass of IRRI104 at maturity was lower but there was no significant difference among genotypes for harvest index. There was no genotype × season interaction for grain yield, aboveground biomass, or harvest index, again suggesting stable performance.

**TABLE 3:** Agronomic performances of elite short‐duration rice genotypes in dry, early wet, and late wet seasons of 2016

Season	Days to heading	Plant height^a^	Grain yield	Aboveground biomass^a^	Harvest index
	d	cm	Mg ha^–1^	g m^–2^	
Genotype					
IR12A165	72	119	5.15	1197	0.38
IR13A378	76	117	5.48	1305	0.40
IR13A438	71	107	5.18	1087	0.40
IRRI104	71	87	3.99	975	0.41
Season					
Dry season	75	101	7.23	1443	0.50
Early wet season	68	122	4.81	1227	0.37
Late wet season	74	99	2.81	753	0.32
ANOVA					
Genotype	ns	**	*	**	ns
Season	**	**	**	**	**
Genotype × season	**	*	ns	ns	ns

a Measured at maturity.

^*^Significant at the .05 probability level.

^**^Significant at the.01 probability level. ns, not significant.

The three promising genotypes had fewer panicles but more spikelets per panicle than IRRI104, although the differences were not significant (Table 4). IRRI104 had significantly smaller grains but the filled grain percentage did not differ significantly among the genotypes. The spikelet sterility was the highest in LWS when the radiation intensity was lowest. IRRI104 had higher sterility than the other genotypes in LWS but there was no significant difference among genotypes in DS, when the radiation intensity was highest.

**TABLE 4:** Yield components of elite short‐duration rice genotypes in dry, early wet, and late wet seasons of 2016

	Panicles	Spikelets per panicle	1000‐grain weight	Filled grains	Spikelet sterility
	m^−2^		g	%	
Genotype					
IR12A165	320	87	28.3	66	24
IR13A378	335	94	27.4	67	25
IR13A438	337	87	26.9	64	31
IRRI104	375	81	25.6	62	32
Season					
Dry season	372	98	27.0	84	15
Early wet season	325	98	27.7	60	31
Late wet season	329	67	26.4	50	37
ANOVA					
Genotype	ns	ns	*	ns	ns
Season	ns	*	ns	**	**
Genotype × season	ns	ns	ns	ns	**

^*^Significant at the.05 probability level.

^**^Significant at the.01 probability level. ns, not significant.

**FIGURE 2:**
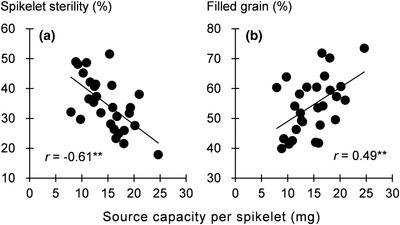
Relationship between source capacity per spikelet and (a) spikelet sterility and (b) filled‐grain percentage for four elite rice genotypes evaluated at the International Rice Research Institute in the early and late wet seasons of 2016. Source capacity is defined as stem nonstructural carbohydrates at heading plus biomass accumulation after heading ^**^ *p* < .01.

Sink capacity did not differ significantly among the genotypes, but dry matter accumulation during grain filling and source capacity were significantly lower for IRRI104 (Table 5). The source/sink ratio and the potential supply of carbohydrates for grain growth (i.e., source capacity per spikelet) were also significantly lower in IRRI104 than in the other genotypes. Again, there was no significant genotype × season interaction, suggesting stable performance. Source capacity per spikelet was significantly negatively correlated with spikelet sterility (*r* = –0.61^**^) and significantly positively correlated with the filled grain percentage (*r* = 0.49^**^; Figure 2).

**TABLE 5:** Physiological traits of sink–source relationships in elite short‐duration rice genotypes in the early and late wet seasons of 2016

	Sink capacity	⊿W^a^	Source capacity	Source‐to‐sink ratio	Source capacity per spikelet
	g m^−2^				mg
Genotype					
IR12A165	683	349	399	0.69	16.6
IR13A378	751	478	512	0.75	17.7
IR13A438	674	322	346	0.61	14.0
IRRI104	678	238	271	0.49	10.8
Season					
Early wet season	753	390	439	0.64	14.8
Late wet season	489	303	325	0.64	14.8
ANOVA					
Genotype	ns	**	**	*	**
Season	**	ns	ns	ns	ns
Genotype × seaon	ns	ns	ns	ns	ns

a Dry matter accumulation during the grain filling stage.

^*^Significant at the .05 probability level.

^**^Significant at the .01 probability level. ns, not significant.

## 4 DISCUSSION

### 4.1 Characteristics of current short‐duration breeding lines in tropical Asia

We observed high variation in grain yield among the genotypes in IRRI's short‐duration rice breeding program, with values ranging from 5.2 to 7.2 Mg ha^−1^ in DS and 6.0 to 8.5 Mg ha^−1^ in EWS (Table 1). IR12A173 achieved the highest mean yield, at 1.8 Mg ha^−1^ higher than IRRI104, and had 6 d more to heading than IRRI104 (Figure 1). These results suggest that there remains considerable room to exploit genetic variation among short‐duration accessions in the factors that lead to high yield. Although a lack of photoperiod sensitivity is a prerequisite for developing short‐duration varieties (Khush et al., 1995), the correlation between days to heading in DS and EWS was not significant (Supplemental Table S1 and Supplemental Table S2; *r* = 0.08, *p* = .359, *n* = 22). This indicates that photoperiod sensitivity varies even among the advanced lines in the breeding program. Although days to heading was significantly correlated with yield, the analysis of covariance detected significant yield differences among the genotypes even after eliminating the effect of days to heading. Plant height may have affected yield in DS but panicle number was not associated with yield.

To identify the physiological characteristics responsible for high yield in the elite short‐duration genotypes, we focused on genotypes with a days to heading value similar to that of IRRI104. IR13A438, IR13A378, and IR12A165 produced 11 to 18% more yield than IRRI104 in 2015 and 29 to 38% higher yield in 2016. These lines were 20 to 32 cm taller than IRRI104. Increasing the height even further is undesirable because the risk of lodging is greater when plant height is more than 120 cm in the tropics (Kato et al., 2019).

### 4.2 Physiological attributes that contributed to high yield in the promising genotypes

Two key growth aspects appear to be responsible for high yield in the short‐duration genotypes. First, high aboveground biomass rather than high harvest index should be the primary breeding target for short‐duration varieties (Table 3). High biomass accumulation in the promising genotypes generally resulted from high mean LAI and, to a lesser extent, from high NAR during the period from heading to maturity (Table 2). The increased plant height in the three promising genotypes was likely to be the reason for higher leaf area at the heading stage and contributed to greater biomass accumulation during the grain‐filling period. Grain yield increased with increased LAI to a maximum of around LAI = 6 in tropical lowland rice, since the plants cannot intercept all the available radiation at lower LAI values (Yoshida & Parao, 1976). However, the LAI of the short‐duration genotypes has not reached this value (Table 2).

The introduction of semidwarfing genes such as *sd1* (Spielmeyer, Ellis, & Chandler, 2002) to modern rice varieties has improved the harvest index (Peng & Khush, 2003). Reduced resource allocation to elongating internodes during the reproductive stage led to less competition between organs for nonstructural carbohydrates, thereby enabling larger panicle size in semidwarf varieties (Kato et al., 2014). Simultaneously, the canopy structure of these varieties has been modified to lower the light extinction coefficient (i.e., by producing more upright leaves and tillers), which has contributed to greater biomass accumulation during the grain‐filling stage (Okami, Kato, & Yamagishi, 2016; Peng et al., 2008). However, breeders of short‐duration varieties should modify plant development to increase the current level of LAI during the limited period of vegetative growth. In addition to the possibility of increasing plant height without semidwarfing genes, increasing the specific leaf area (leaf area divided by leaf weight) and the leaf weight ratio (leaf weight/total plant weight) could theoretically increase LAI (Kropff et al., 1994). Previous studies suggested large genotypic variation in these traits (Dingkuhn, Johnson, Sow, & Audebert, 1999; Okami, Kato, & Yamagishi, 2012).

The second key growth aspect appears to be that low source capacity during grain filling, rather than low sink capacity, is the major cause of IRRI104's low yield. IRRI104 tended to have a lower filled grain percentage than other genotypes, partly because of its high spikelet sterility (Table 4). This can be attributed to the limited carbohydrate availability for grain growth (Figure 2). On the other hand, the high‐yielding genotypes had a higher source capacity than IRRI104, leading to a higher source‐to‐sink ratio and a higher source capacity per spikelet (Table 5). The higher source/sink ratio can mainly be attributed to the increased biomass accumulation during the grain‐filling stage. Compared with IRRI104, increasing plant height with slightly reduced panicle number increased the leaf area of promising short‐duration genotypes (Supplemental Table S3). Accordingly, we suggest that the increased plant height and LAI in the elite genotypes increased photoassimilation during the grain‐filling stage, leading to higher grain yield. Although sink capacity did not differ significantly among the genotypes (Table 5), breeding to increase panicle size, thereby increasing the number of spikelets m^−2^ and sink capacity, should further improve the grain yield of these genotypes. However, modification of panicle morphology is often accompanied by changes in grain shape and size (Ohsumi et al., 2011) and caryopsis development (Ishimaru, Matsuda, Ohsugi, & Yamagishi, 2003), which may affect grain qualities such as chalkiness. These hypotheses should be tested in future research on short‐duration rice genotypes.

In the 1960s, the introduction of semidwarf plant types with a large number of tillers dramatically increased the maximum LAI of the modern medium‐duration varieties used in tropical Asia (Yoshida & Parao, 1972). Although this study did not compare short‐ and medium‐duration varieties, the relevance of plant height (Table 3) and the irrelevance of panicle number (Table 4) for LAI around the heading stage (Table 2) suggest that the optimal ratio of stem size (leaf area per stem) to stem number may be higher for short‐duration varieties than for medium‐duration varieties. If that suggestion is correct, the increase in spikelets m^−2^ must be achieved not by increasing panicle number but by increasing panicle size. In fact, our previous study on elite short‐duration rice varieties in central China suggested the importance of panicle size for high yield (Xu et al., 2018), although the cause and effect relationship remained unclear. This study suggests that the ideotype for short‐duration varieties differs from that for medium‐duration varieties (Peng et al., 2008). Intermediate plant height may be desirable for short‐duration varieties, but semidwarf for medium‐duration varieties. Future research would clarify whether modifying the plant type towards greater leaf area while decreasing the panicle number is causally associated with high yield in the promising genotypes.

## 5 CONCLUSIONS

In this study, we identified three promising short‐duration rice varieties from the IRRI rice breeding program and investigated the factors responsible for their high yield. These accessions produced yields 11 to 38% higher than that of IRRI104, which is currently the most popular short‐duration variety in Southeast Asia. These genotypes were 20 to 32 cm taller than IRRI104, suggesting that the semidwarf ideotype pursued for medium‐duration rice varieties may not be the ideal form. We also found that low source capacity for grain filling was the major cause of the relatively low yield of IRRI104. The promising genotypes had significantly higher source/sink ratios than IRRI104, which could be attributed mainly to the higher LAI that supported greater biomass accumulation during the grain‐filling stage. We suggest that it may be beneficial to modify plant development to have intermediate plant height and permit greater leaf area expansion during the vegetative stage for short‐duration rice varieties in tropical Asia.

## CONFLICT OF INTEREST DISCLOSURE

The authors declare that there is no conflict of interest.

## Supporting information


**Supplemental Table S1**. Agronomic performances of 36 advanced short‐duration rice genotypes at the IRRI farm during the dry season (January–April) of 2015.
**Supplemental Table S2**. Agronomic performances of 36 advanced short‐duration rice genotypes at the IRRI farm during the early wet season (June–September) of 2015.
**Supplemental Table S3**. Summary of the growth characteristics of three promising short‐duration rice genotypes in comparison with IRRI104 across three seasons in 2016
