# Supplementary material for: Identification and characterization of high‐yielding, short‐duration rice genotypes for tropical Asia
Source: Crop Sci. 2020 Aug 3;60(5):2241–50. doi: 10.1002/csc2.20183 (PMC7689942; doi:10.1002/csc2.20183)
Supplement: Supplementary file 1 — Supplemental Table S1. Agronomic performances of 36 advanced short‐duration rice genotypes at the IRRI farm during the dry season (January–April) of 2015. Supplemental Table S2. Agronomic performances of 36 advanced short‐duration rice genotypes at the IRRI farm during the early wet season (June–September) of 2015. Supplemental Table S3. Summary of the growth characteristics of three promising short‐duration rice genotypes in comparison with IRRI104 across three seasons in 2016 [file CSC2-60-2241-s001.docx]

| **Supplementary Table S1.** Agronomic performances of 36 advanced short-duration rice genotypes at the International Rice Research Institute during the dry season (January to April) 2015. | | | | |
| --- | --- | --- | --- | --- |
| Genotype | Days to heading  (d) | Grain  yield  (t ha^-1^) | Plant  height  (cm) | Panicles  (m^-2^) |
| IR13A438 ^a^ | 78 | 6.94 | 99 | 323 |
| IR07A253 ^a^ | 80 | 5.95 | 95 | 369 |
| IR09N536 ^a^ | 80 | 6.27 | 88 | 393 |
| IR99054-B-B-31 ^a^ | 80 | 5.54 | 96 | 278 |
| IR12A248 ^a^ | 80 | 6.04 | 96 | 359 |
| IR11N121 ^a^ | 80 | 6.80 | 93 | 353 |
| IRRI123 ^a^ | 80 | 6.15 | 87 | 369 |
| IR13A157 | 80 | 5.64 | 89 | 294 |
| IR99090-B-B-59 ^a^ | 80 | 6.08 | 92 | 388 |
| IR99065-B-B-6 | 81 | 6.62 | 99 | 299 |
| IR09A130 ^a^ | 81 | 6.66 | 89 | 395 |
| IR13A347 | 81 | 5.41 | 90 | 282 |
| IR09A128 ^a^ | 82 | 6.05 | 98 | 354 |
| IR13A387 ^a^ | 82 | 5.95 | 91 | 340 |
| IR99084-B-B-44 | 82 | 6.44 | 102 | 322 |
| IRRI104 ^a^ | 82 | 5.97 | 88 | 408 |
| IR12N125 ^a^ | 82 | 6.45 | 94 | 387 |
| IR13A378 ^a^ | 82 | 5.92 | 100 | 314 |
| IR12A165 ^a^ | 82 | 6.50 | 110 | 398 |
| IR07A107 ^a^ | 83 | 6.22 | 91 | 343 |
| IR08N150 ^a^ | 83 | 5.23 | 99 | 318 |
| IR09N516 ^a^ | 83 | 6.50 | 87 | 338 |
| IR10A108 | 83 | 5.92 | 91 | 331 |
| IR12A200 ^a^ | 84 | 6.80 | 114 | 293 |
| IR11A208 ^a^ | 84 | 6.40 | 93 | 362 |
| IR14N109 ^a^ | 84 | 6.45 | 84 | 313 |
| IR12A173 ^a^ | 84 | 7.17 | 107 | 292 |
| IR99092-B-B-53 | 85 | 6.72 | 93 | 313 |
| IR14A201 | 85 | 6.52 | 92 | 308 |
| IR13A185 | 86 | 6.40 | 96 | 279 |
| IR14A133 | 87 | 6.08 | 92 | 309 |
| IR13A293 | 88 | 6.82 | 95 | 324 |
| IR12A229 | 89 | 6.66 | 92 | 283 |
| IR12N191 | 91 | 7.12 | 109 | 308 |
| IR12N245 | 93 | 6.67 | 96 | 270 |
| IR12N260 | 96 | 6.69 | 106 | 237 |
| LSD (0.05) | 4 | 0.95 | 11 | 64 |
| LSD; least-significant difference. a; genotypes evaluated in both dry and early wet seasons of 2015. | | | | |

| **Supplementary Table S2.** Agronomic performances of 36 advanced short-duration rice genotypes at the International Rice Research Institute during the early wet season (June to September) 2015. | | | | |
| --- | --- | --- | --- | --- |
| Genotype | Days to heading  d) | Grain  yield  (t ha^-1^) | Plant  height  (cm) | Panicles (m^-2^) |
| IR13A387 ^a^ | 75 | 5.97 | 109 | 484 |
| IR09N536 ^a^ | 76 | 6.59 | 97 | 495 |
| IRRI104 ^a^ | 76 | 6.04 | 95 | 487 |
| IR99081-B-B-B-35 | 76 | 6.87 | 123 | 468 |
| IR13A438 ^a^ | 77 | 6.36 | 108 | 473 |
| IR10A136 | 77 | 8.04 | 113 | 600 |
| IR10N238 | 77 | 6.92 | 131 | 428 |
| IR99076-B-B-6 | 77 | 7.59 | 125 | 425 |
| IRRI175 | 77 | 6.82 | 134 | 460 |
| IR07A253 ^a^ | 78 | 6.79 | 98 | 520 |
| IR13A378 ^a^ | 81 | 7.44 | 123 | 466 |
| IR12A248 ^a^ | 81 | 6.76 | 122 | 431 |
| IR12A165 ^a^ | 82 | 7.65 | 128 | 425 |
| IR12A200 ^a^ | 83 | 7.18 | 137 | 396 |
| IR98413-B-B-1 | 84 | 7.97 | 113 | 433 |
| IR12A173 ^a^ | 84 | 8.39 | 118 | 384 |
| IR11A208 ^a^ | 85 | 7.44 | 114 | 495 |
| IR99090-B-B-59 ^a^ | 85 | 5.96 | 111 | 502 |
| IR07A107 ^a^ | 85 | 7.61 | 109 | 429 |
| IR09N516 ^a^ | 86 | 7.58 | 106 | 511 |
| IR98418-B-B-2 | 86 | 6.16 | 130 | 490 |
| IR11N121 ^a^ | 87 | 7.76 | 115 | 463 |
| IR08N150 ^a^ | 87 | 8.19 | 117 | 423 |
| IR09A130 ^a^ | 87 | 7.62 | 113 | 471 |
| IR100004-87-B | 87 | 7.50 | 120 | 485 |
| IR100740-26-B-2 | 87 | 7.50 | 96 | 558 |
| IR10M239 | 87 | 7.43 | 125 | 528 |
| IR11A106 | 87 | 7.11 | 122 | 585 |
| IR103806-517-BRGA | 88 | 6.88 | 113 | 523 |
| IR99112-B-B-9 | 88 | 8.54 | 121 | 583 |
| IR09A128 ^a^ | 88 | 7.95 | 120 | 472 |
| IR100008-91-B | 88 | 7.14 | 113 | 553 |
| IR12N125 ^a^ | 88 | 8.08 | 112 | 418 |
| IR14N109 ^a^ | 88 | 6.66 | 103 | 469 |
| IRRI123 ^a^ | 89 | 7.88 | 114 | 433 |
| IR99054-B-B-31 ^a^ | 90 | 7.11 | 137 | 381 |
| LSD (0.05) | 3 | 1.28 | 13 | 74 |
| LSD; least-significant difference. a; genotypes evaluated in both dry and early wet seasons of 2015. | | | | |

| **Supplementary Table S3.** Summary of the growth characteristics of three promising short-duration rice genotypes in comparison to IRRI104 across three seasons in 2016. | | | |
| --- | --- | --- | --- |
|  | Panicle number (m^-2^)^a^ | Plant height  (cm)^b^ | Leaf area index during the reproductive stage (m^2^ m^-2^)^c^ |
| IR12A165 | 320 | 119 | 3.02 |
| IR13A378 | 335 | 117 | 2.79 |
| IR13A438 | 337 | 107 | 2.74 |
| IRRI104 | 375 | 87 | 2.58 |
| a; adopted from Table 4, b; adopted from Table 3, c; adopted from Table 2. | | | |
